# Neurobehavioral Toxicity of a Repeated Exposure (14 Days) to the Airborne Polycyclic Aromatic Hydrocarbon Fluorene in Adult Wistar Male Rats

**DOI:** 10.1371/journal.pone.0071413

**Published:** 2013-08-20

**Authors:** Julie Peiffer, Frédéric Cosnier, Nathalie Grova, Hervé Nunge, Guillaume Salquèbre, Marie-Josèphe Decret, Benoît Cossec, Guido Rychen, Brice M. R. Appenzeller, Henri Schroeder

**Affiliations:** 1 Unité de Recherche Animal et Fonctionnalités des Produits Animaux, INRA UC340, Université de Lorraine, Vandœuvre-lès-Nancy, France; 2 Unité de Services Inter-Laboratoires, Département Polluants et Santé, Institut National de Recherche et de Sécurité, Vandœuvre-lès-Nancy, France; 3 Laboratory of Analytical Human Biomonitoring, CRP-Santé, Luxembourg Ville, Luxembourg; University of Insubria, Italy

## Abstract

Fluorene is one of the most abundant polycyclic aromatic hydrocarbons in air and may contribute to the neurobehavioral alterations induced by the environmental exposure of humans to PAHs. Since no data are available on fluorene neurotoxicity, this study was conducted in adult rats to assess the behavioral toxicity of repeated fluorene inhalation exposure. Male rats (n = 18/group) were exposed nose-only to 1.5 or 150 ppb of fluorene 6 hours/day for 14 consecutive days, whereas the control animals were exposed to non-contaminated air. At the end of the exposure, animals were tested for activity and anxiety in an open-field and in an elevated-plus maze, for short-term memory in a Y-maze, and for spatial learning in an eight-arm maze. The results showed that the locomotor activity and the learning performances of the animals were unaffected by fluorene. In parallel, the fluorene-exposed rats showed a lower level of anxiety than controls in the open-field, but not in the elevated-plus maze, which is probably due to a possible difference in the aversive feature of the two mazes. In the same animals, increasing blood and brain levels of fluorene monohydroxylated metabolites (especially the 2-OH fluorene) were detected at both concentrations (1.5 and 150 ppb), demonstrating the exposure of the animals to the pollutant and showing the ability of this compound to be metabolized and to reach the cerebral compartment. The present study highlights the possibility for a 14-day fluorene exposure to induce some specific anxiety-related behavioral disturbances, and argues in favor of the susceptibility of the adult brain when exposed to volatile fluorene.

## Introduction

Polycyclic aromatic hydrocarbons (PAHs) are persistent organic pollutants which are ubiquitously distributed in air, food, soil, and various occupational settings. Whereas the ingestion of PAH-contaminated food products appears as the dominant source of exposure, non-occupational inhalation of polluted air could be of great concern in places associated with a high population density level and/or a consistent rate of industrialization. Based on the findings of several studies [1], [2], the non-occupational levels of PAH exposure are comprised between 1 and 440 ng/m^3^ depending on the location of the study, the location of the sampling sites, the season of sampling, the phase investigated (gas and/or particle phases), and the number of PAHs measured in air. PAHs are also well-known as a source of occupational exposure for significant groups of workers with levels that can reach several tens of µg/m^3^ [3], [4].

PAHs are identified as toxic since a number of them have potent mutagenic, carcinogenic and endocrine disrupting chemical properties [3], [5], [6]. In contrast, the potential neurotoxicity of PAHs has received much less attention despite some human and animal evidence (for review, see reference [7]). In humans, PAH-related neurological symptoms were reported in residents living near two dumping sites in Texas [8] or a waste oil reprocessing plant in California [9], and in workers of a coke processing plant in Poland [10]. More recently, the occupational exposure of 178 coke oven workers to benzo(a)pyrene (BaP) was demonstrated to induce alterations of both emotional and cognitive functions correlated with significant decreases in monoamine, amino acid and choline neurotransmitter levels [11]. In parallel, studies performed in adult animals showed the possibility for acutely administered BaP and fluoranthene to disrupt some behavioral endpoints including motor activity, responsiveness to sensory stimuli, and physiological and autonomic responses in correlation with the brain concentrations of both compounds [12], [13]. Cognitive and anxiety-related disorders were also demonstrated in adult mice sub-acutely treated with increasing doses of BaP (0.02 to 200 mg/kg/day, 10 days, i.p.) [14], [15]. Such results were associated with concomitant dose-related changes in the expression of the NR1 and NR2A subunits of the NMDA glutamate receptor in regions involved in memory and anxiety. Taken all together, these observations have led to speculations about a possible relationship between neurobehavioral alterations and exposure to environmental PAHs [7], [16].

Therefore, the purpose of the present study was to develop a rodent model of PAH inhalation exposure in order to investigate the neurobehavioral toxicity of such chemicals. Among the PAHs, fluorene was used as a representative compound for the following reasons: 1) fluorene is highly volatile with air concentration being one of the most abundant [1], [5], [17], 2) it is used as intermediate chemical in various industrial applications, e.g., production of resins, dye stuffs, drugs and other chemical products [5], 3) it is found in many environmental samples including food, tobacco smoke and industrial waste [5], [18], [19], and 4) fluorene is listed among the 16 PAH priority pollutants for human health by the WHO and the US-EPA [20]. Consequently, fluorene can be considered a relevant contaminant from both occupational and general PAH atmospheric exposure. In the present study, adult male rats were assessed for their behavioral performances related to locomotor activity, learning and memory, and anxiety, after 14-day repeated inhalation of fluorene. In parallel, behavioral and physiological responses to the stressful context of the model of exposure used (exposure nose-only by placing the animals into restrained tubes) were measured as a means of inferring the magnitude of the stress response from the behavioral toxicity induced by fluorene, as suggested by the 403 OECD guideline for inhalation studies [21]. Finally, analyses of fluorene and three of its hydroxylated metabolites were performed in plasma and brain samples in order to ensure the exposure of the animals to this contaminant.

## Materials and Methods

### 1. Animals

Adult Wistar Han male rats weighing 225–250 g (8–9 weeks of age) were purchased from Harlan (Gannat, France). They were housed in a regulated environment (22±3°C; humidity 55±10%; light on from 3∶00 a.m. to 3∶00 p.m.) and subjected to a 7-day acclimatization period before study. Food (Teklad Global Diet 2016, Harlan, Gannat, France) and tap water were provided *ad libitum*. Atmospheric air was used to ventilate all the rooms of the animal house. To this end, it was filtered for particles over 0.1 µm of diameter and adjusted to a temperature of 22±1°C and humidity of 55±10 %. All rats were handled in the same way and were randomly allocated to experimental groups. All procedures were in compliance with the rules provided by the European Union (Directive 2010/63/EU) [22], and were approved and supervised by the institutional ethics committee of the University of Lorraine (authorization number A 54–547–13).

### 2. Experimental design

Rats (n = 18/group) were exposed nose-only to fluorene (purity ≥99%, Sigma Aldrich, St-Quentin-Fallavier, France) vapor for a daily period of 6 hours over 14 consecutive days. Two levels of contamination were studied: 1.5 and 150 ppb (10 µg/m^3^ and 1 mg/m^3^), whereas the control animals were exposed to clean air for the same time. The daily exposure period was limited to 6 hours in order to respect the illuminated part of the circadian cycle and to allow the behavioral testing during the dark part of the cycle within the same day. Thus, considering this period of time and the air volume inhaled by the rats (12 L/hour), the lowest concentration of fluorene was calculated in order to reach in the animals similar levels of exposure to those observed in humans living in urban areas [1], [2] whereas the highest one was more characteristic of occupational exposure [4], [11]. At the end of the exposure period, 12 animals from each group were used for behavioral investigations whereas the remaining 6 rats were sacrificed for biological measurements.

### 3. Fluorene inhalation exposure

Inhalation was conducted in accordance with the 403 OECD guideline for inhalation studies [21] in 200 L glass/stainless-steel chambers equipped with clear non-vented inhalation tubes made of polycarbonate (length: 25 cm, internal diameter: 5.5 cm) that ensure exposure through the nasal way and allow the tail of the rat to remain outside for natural thermoregulation. Designed to sustain a dynamic and adjustable airflow (4–5 m^3^/h), the chambers were maintained at a negative pressure of no more than 5 mm H_2_O in order to prevent any leakage of the atmosphere. Ambient air was used to ventilate all the chambers and was pretreated under the same conditions as those applied to the animal house (for details, see the 1^st^ paragraph of the materials and methods section). After conditioning for temperature and humidity, the input atmosphere was contaminated by passing the total airflow over a thermoregulated solid bed of fluorene, and was checked to ensure the total absence of aerosol under these conditions of generation by using a pDR-1200 aerosol monitor (particle sizing range: 1–10 µm, Thermo Electron Corporation, Waltham, MA, USA). Thus, fluorene contained in the atmosphere was exclusively under gaseous form. Each period of inhalation was monitored for air fluorene concentrations, temperature and relative humidity, as recommended by the 403 OECD guideline for inhalation studies [21].

### 4. Atmosphere sampling and analysis

Fluorene atmospheric concentrations were determined twice during the 6-hour period for 1.5 ppb, and six times for 150 ppb, by collecting atmosphere samples through glass tubes packed with Amberlite XAD-2 Polymeric Adsorbent. Sampling frequencies were adapted to allow the collection of an adequate volume of air for the quantification of fluorene (150 L at 1.5 ppb and 50 L at 150 ppb) above the limit of quantification of the method. Thus, fluorene was desorbed with acetonitrile containing anthracene as internal standard (400 mg/L) and then analyzed on a Varian CP-3800 gas chromatograph equipped with a flame ionization detector. Samples were separated on a 30 m×0.53 mm (1.5 µm film thickness) CP-Sil 5 column, using nitrogen as carrier gas. The flow rate was 4 mL/min. The column temperature program was 190°C for 4 min, increased to 235°C at a rate of 15°C/min and held for 2 min. The injector (flash 1061) and the detector were maintained at 240°C and 250°C, respectively. The limit of quantification of the method was 2.50 µmol/L in acetonitrile that corresponds to a fluorene air concentration of 8.3 µg/m3 (1.25 ppb). Atmosphere samples of the two control chambers were also collected over the same period under the same sampling conditions (two samples for the 6-hour period) to ensure the absence of contamination from the air used. In parallel, temperature and humidity were measured within the 4 chambers one time/min over the 6 hour-period of exposure through the appropriate probes.

### 5. Brain and plasma sampling and analysis

#### 5.1. Instrumentation

Analyses were carried out with an Agilent 7890A gas chromatograph equipped with a HP-5MS capillary column (30 m, 0.25 mm i.d., 0.25 µm film thickness), coupled with an Agilent 7000A triple quadrupole mass spectrometer operating in electron impact ionization mode and an Agilent CTC PAL autosampler. Details of analytical conditions used for chromatography and MS/MS detection were previously described [23].

#### 5.2. Blood analysis

Blood was collected in heparinized tube by tail venous ponction at the end of the last exposure period, centrifuged for 3 min at room temperature, and plasma was stored at −20°C until further use. Samples were supplemented with 20 µL of internal standard mix solution (0.1 mg/mL) of fluorene*-d10* and naphthol*-d7* as internal standards and adjusted to pH 5.7 with 200 µL of 1M sodium acetate buffer. The hydrolysis, extraction and purification procedures were similar to those described for brain analysis (see the following paragraph). Plasma extracts were reconstituted in 25 µL of MSTFA N-methyl-N-(trimethylsilyl) trifluoroacetamide, Sigma-Aldrich, Bormen, Belgium). The analytes were derivatized for 30 min at 60°C and then 2 µL were injected into the GC-MS/MS. Calibration curves were performed using plasma specimens supplemented with increasing added concentrations of fluorene and 2-, 3- and 9-OH fluorene from 0 to 250 ng/mL of plasma. LODs were ranged between 0.05 and 0.1 ng/mL and LOQs between 0.15 and 0.3 ng/mL for both fluorene and monohydroxylated metabolites.

#### 5.3. Brain analysis

The rats were sacrificed by decapitation at the end of the last exposure period. Thus, the whole brain was immediately removed, frozen in liquid nitrogen and stored at −80°C until further use. The determination of fluorene and 2-, 3- and 9-OH fluorene in brain was performed in accordance with the analytical method previously described by Grova et al. (2011) [23]. Brain samples (100 mg) were homogenized with 200 µL of ultrapure H_2_O, 0.1% of triton X-100 and 20 µL of internal standard mix solution (0.1 mg/mL) of fluorene*-d_10_* and naphthol*-d_7_* (MRI/NCI, Kansas City, Missouri, USA). Sodium acetate buffer (pH 5.6, 1 M) was added and hydrolysis was performed overnight at 37°C using 5 µL of sulfatase (10 units/µL) and 5 µL of glucuronidase (127 units/µL) from *Helix pomatia* (Sigma-Aldrich, Bormen, Belgium). A first extraction was carried out with ethyl acetate/cyclohexane (EA/CH) (50∶50, v/v). The supernatant was collected and solvents were evaporated under N_2_ at 37°C. The residue was dissolved into CH and applied onto an Envi-Chrom P SPE column (Sigma-Aldrich, Bormen, Belgium) previously conditioned with CH. Fluorene and OH-fluorenes were eluted with EA/CH (50∶50, v/v) and then evaporated. The residues were dissolved in 2 mL CH, 1.6 mL methanol and 0.4 mLwater (50∶40∶10, v/v/v) and the two layers were separated. The CH layer containing the fluorene was dried under N_2_ and submitted to saponification for 1 h at 60°C (1 mL alcoholic KOH 7%, w/v). Then, 2 mL of ultrapure water were added to 2 mL of EA/CH (50∶50, v/v). Finally, the upper phase containing the fluorene was collected and dried under N_2_ until 50 µL. Thus, 1 µL was injected into the GC-MS/MS. In parallel, the methanol-water layer containing OH-fluorenes was evaporated to dryness and the extract was reconstituted in 40 µL of MTBSTFA (N-Methyl-N-(t-butyldimethylsilyl) trifluoroacetamide, 1% t-BDMCS, ≥97% purity, Sigma-Aldrich, Bormen, Belgium). The analytes were derivatized for 30 min at 60°C and then 2 µL were injected into the GC-MS/MS. Calibration curves were performed using brain specimens supplemented with increasing concentrations of fluorene and 2-, 3- and 9-OH fluorene from 0 to 500 ng/g of brain tissue. LODs were evaluated at 1.9 ng/g for fluorene and ranged between 0.10 and 0.25 ng/g for OH-fluorenes in brain. LOQs were determined at 6.3 ng/g of brain tissue for fluorene and ranged between 0.50 and 0.80 ng/g for OH-fluorenes.

### 6. Stress assessment

In accordance with the recommendations of the 403 OECD guideline for inhalation studies [21], two kinds of verification were performed in the experimental design in order to discriminate between the stress-related effects induced by the model of exposure and those of the contaminant: 1) two control groups were used for the present study, one being exposed through the inhalation tubes and the other one being placed within the chamber in small wirecloth enclosures that allow them to move freely in the cages, and 2) rats from the restraint-control group and the two fluorene-exposed groups were familiarized by being placed them into the tube for 6 hours/day for the previous 8 days before starting the exposure. Then, variations of stress-related markers including weights of brain, liver and surrenal gland were recorded after 14 days of exposure in the 4 groups of animals whereas body weight and blood corticosterone levels were measured prior to the daily 6 h exposure at 4 predefined stages of the experimental procedure, i.e., before starting the habituation to the restraint tubes, before starting the exposure, and after 7 and 14 days of fluorene inhalation. At each stage and for each rat, blood tail samples were collected into heparinized tubes, centrifuged for 3 min at room temperature, and plasma was stored at −20°C.

### 7. Corticosterone assay

Corticosterone blood levels were determined using a high-performance liquid chromatography (HPLC) method adapted from Ling and Jamali (2003) [24]. Briefly, 1 mL of ethyl acetate containing 50 µg/L of betamethasone (Sigma Aldrich, St-Quentin-Fallavier, France) as internal standard was added to 100 µL of plasma. The mixture was shaken vigorously for 30 s and centrifuged at 1800×g for 10 min. The supernatant was collected, rinsed with 1 mL of NaOH 0.1 M, mixed for 30 s and centrifuged for 10 min at 1800×g. Then, it was removed again and evaporated until dryness. The final residue was dissolved in 300 µL of mobile phase and filtered through a 0.45 µm filter. A volume of 100 μL was injected in a Lichrospher 100 RP-18 column (150×2 mm I.D., 5 µm particle size, C.I.L. Cluzeau, Sainte Foy la Grande, France). The HPLC system consisted of a Waters Alliance 2690 system coupled with a Waters 996 diode array detector. The column temperature was set at 40°C. A water/acetonitrile solution (70∶30, v/v) was used as the mobile phase at a flow rate of 0.25 mL/min. Corticosterone was detected at 245 nm wavelength and quantified over a calibration curve (corticosterone, >99% purity, Sigma Aldrich, St-Quentin-Fallavier, France) spiked with betamethasone. Thus, corticosterone blood levels were calculated by their peak area ratio with the internal standard. The standard curve was obtained by injecting the same volume of solutions containing increasing amounts of corticosterone (3.5, 8.75, 17.5, 35 and 52.5 ng/mL) spiked with the same quantity of betamethasone. All the standard solutions were dissolved into the mobile phase and subjected to the same extraction procedure.

### 8. Behavioral testing

All the behavioral testing was performed within one hour of the beginning of the dark phase of the cycle, i.e., at 4∶00 p.m. In each test, behavior of all the animals was videotaped and variables quantified by the same experienced researcher using The Observer XT v8.0 software (Noldus, Wageningen, The Netherlands).

#### 8.1. Elevated-plus maze

Anxiety levels were assessed in the elevated-plus maze referred to the protocol described by Violle et al. (2009) [25]. The maze consists of two open arms (50×10 cm, length × width) and two enclosed arms (50×10×50 cm, length × width × height) that are joined by a central platform (10×10 cm, length × width) to form a plus shape. The apparatus was 70 cm above the floor. Rats were placed in the central area, facing an open arm, and allowed to freely explore both parts of the maze over a 5 min period. Standard behavioral measurements including the number of open and closed arm entries, and the time spent in both sections of the maze were quantified. Additional ethological variables related to risk assessment and exploration (number of head-dipping and rears) were also investigated [25].

#### 8.2. Open-field

Activity was assessed in a circular open-field platform (diameter 1 m, maze divided in 32 squares). After a 1-min adaptation period, rats were placed individually into the maze, and their activity videotaped for 5 min. Results were expressed as the total number of squares crossed, and the number of squares visited and the time spent in both peripheral, intermediate or central areas of the maze [26].

#### 8.3. Y-maze

Immediate working memory performance was investigated by recording spontaneous alternation behavior in a Y-maze formed of 3 arms (60×10×45 cm, length × width × height) positioned at equal angles [27]. The rats were placed at the end of one arm and allowed to freely explore the maze for 10 min. The series of arm entries was recorded and alternation calculated as successive entries into the 3 arms on overlapping triplet sets. The alternation percentage was calculated as the ratio of actual to possible alternations (defined as the total number of arm entries minus two, multiplied by 100). Otherwise, the number of arm entries and the number of rears were measured as indicators of activity.

#### 8.4. Eight-arm maze

Rats were tested in the eight-arm maze referred to the procedure described by Blaise et al. (2009) [28]. The apparatus consists of 8 projected arms (60×10×10 cm, length × width × height) each of a circular central area measuring 30 cm in diameter, and extra-maze spatial cues consisting of 3 distinct geometrical signs were placed on the walls of the testing room to allow orientation in the maze. Then, rats were tested for their spatial learning abilities once daily for 12 consecutive days. Before each session, every arm was baited with a 15 mg food pellet. Then, rats were left to explore the maze until they had either visited all eight arms or for a 15 min elapsed time. The time and sequence of arm entries, and the number of errors (that corresponds to a re-entry into an already visited arm) were measured during each session. Prior to testing, rats were submitted for 3 days to one session (15 min) per day with a large amount of pellets by the end of each arm to get them accustomed to the maze.

### 9. Statistical analysis

All results are expressed as mean ± S.E.M. Temperature, humidity and fluorene atmospheric concentrations were compared to the appropriate reference value using a one-sample t test. The effects of fluorene were analyzed using a one-way analysis of variance followed by a post-hoc Dunnett t-test with the restrained control group used as reference. The results obtained from the eight-arm maze were analyzed using a two-way analysis of variance with repeated measures on one factor. In addition, behavioral and physiological variables measured in restraint animals were compared to those of free-exposed rats by means of a Student t-test. The statistical analysis was performed using the SPSS 16.0 for Windows software (SPSS Inc., Chicago, IL., USA). Differences were considered significant at the level of *p*<0.05.

## Results

### 1. Atmospheric concentrations of fluorene

Over the whole period of exposure, temperature and humidity measured within the 4 chambers did not significantly differ from the target values (Table 1), and were in compliance with the limits allowed by the 403 OECD guideline for inhalation studies (22±3°C for temperature and 55±10% for humidity) [21]. According to the day of exposure, the concentration of fluorene in the air chamber was between 1.30±0.09 and 1.60±0.08 ppb for the lowest level of exposure, and between 144.3±2.0 and 157.2±2.5 ppb for the highest one. These concentrations did not significantly differ from the target values of 1.5 and 150 ppb during the 14 days of exposure, except for the ones measured during the day 1 and the day 4 of exposure in the 150 ppb contaminated group (Table 1). In parallel, the monitoring of air samplings in the control chambers did not show any detectable levels of fluorene (Table 1).

**Table 1:** Atmospheric metrology of the daily fluorene exposure.

Variable	Freely- moving rats	Restrained controls	1.5 ppb	150 ppb
Fluorene (ppb)				
day 1	n.d.	n.d.	1.43±0.06	157.2±2.5 **
day 4	n.d.	n.d.	1.42±0.03	144.3±2.0 **
day 7	n.d.	n.d.	1.30±0.09	146.1±2.2
day 10	n.d.	n.d.	1.63±0.08	152.4±2.3
day 14	n.d.	n.d.	1.54±0.06	151.7±2.4
Temperature (°C)				
day 1	22.7±0.2	22.2±0.2	21.9±0.1	22.2±0.2
day 4	22.7±0.2	22.1±0.2	21.9±0.3	22.2±0.3
day 7	22.7±0.2	22.1±0.2	21.9±0.3	22.2±0.3
day 10	22.7±0.2	22.2±0.2	21.9±0.2	21.8±0.1
day 14	22.9±0.2	22.3±0.1	22.0±0.1	22.2±0.2
Humidity (%)				
day 1	56.9±1.7	54.4±2.6	58.4±2.5	57.9±2.4
day 4	56.8±1.6	55.5±3.0	56.0±3.1	58.8±1.5
day 7	55.5±0.3	53.4±1.6	49.0±1.8	55.6±2.1
day 10	58.3±3.2	55.0±3.3	50.7±3.3	58.1±5.2
day 14	55.8±0.4	57.6±4.2	59.0±3.1	59.3±4.3

Only the results of 5 days of exposure chosen during all the period of 14 days are presented. The results are expressed as mean±S.E.M. calculated from the values obtained through the 6 hour-period of exposure and were compared to a test value of 22°C for temperature, 55% for humidity, and 1.50 or 150 ppm of fluorene by means of a one-sample t test. ** p<0.01, statistical significant difference from the appropriate test value.

### 2. Blood levels of fluorene and mono-hydroxylated metabolites

Fluorene showed mean concentrations which ranged between 73±11 ng/mL and 71±23 ng/mL of plasma for both control groups. The levels of fluorene detected in the plasma of exposed animals at 1.5 ppb and 150 ppb decreased compared to the control values but they were not statistically different (Table 2). Although the 9-OH fluorene was already measured in both control groups (freely-moving and restrained rats) and 1.5 ppb-exposed rats, a significant increase was observed between these 3 groups and the highest level of exposure (150 ppb). The 3- and the 2-OH fluorene, which were not detected in all the control rats, were quantified in the plasma of both 1.5 ppb- and 150 ppb-exposed groups. The 3-OH fluorene was detected in the plasma of rats exposed to 1.5 ppb with a concentration that was between the LOD and the LOQ. Thus, this concentration was 50-fold increased in 150 ppb-treated animals (p<0.01, Table 2). Concomitantly, the concentration of 2-OH-fluorene in plasma was 10 times higher than the LOQ (0.15 ng/mL) in rats exposed to fluorene (1.5 ppb) and showed a 100-fold increase in 150 ppb-treated animals (Table 2). Moreover, an additional peak was detected at retention time 6.52 min in the plasma from rats exposed to 150 ppb of fluorene. This peak corresponds to the transitions common to OH-fluorene with a precursor ion at m/z = 239.1 and a product ion at m/z = 165. Given that the retention times for the 2-, 3- and 9-OH-fluorene isomers were identified, this peak is likely to correspond to the 1- or the 4- monohydroxylated isomers.

**Table 2:** Blood and brain concentrations of fluorene and monohydroxylated metabolites.

Variable	Freely-moving rats	Restrained controls	1.5 ppb	150 ppb	ANOVA	
					F(3, 20)	*p*
**Blood (ng/mL)**						
Fluorene	72.6±11.3	70.8±22.5	56.4±22.3	35.6±8.9	0.904	n.s.
9-OH fluorene	0.98±0.13	1.42±0.21	0.75±0.21	3.80±0.46 *	27.007	<0.01
3-OH fluorene	n.d.	n.d.	< LOQ	2.48±0.57 **	22.335	<0.01
2-OH fluorene	n.d.	n.d.	1.10±0.15 **	105.0±18.4 **	39.095	<0.01
**Brain (ng/g tissue)**						
Fluorene	34.121±2.38	31.97±2.28	21.13±3.68 **	19.82±0.31 **	8.763	<0.01
9-OH fluorene	0.68±0.06	0.76±0.26	0.54±0.02	1.35±0.23 *	4.116	<0.05
3-OH fluorene	n.d.	n.d.	n.d.	n.d.	–	–
2-OH fluorene	n.d.	n.d.	< LOQ	0.55±0.09 **	24.417	<0.01

Results are expressed as mean ± S.E.M. of n = 6 rats/group. Dunnett's t-test was used for multiple comparisons. ** *p*<0.01, * *p*<0.05, statistical significant differences from restrained controls. Abbreviations: n.d., non detectable, n.s., non significant, LOQ, limit of quantification.

### 3. Brain levels of fluorene and mono-hydroxylated metabolites

Fluorene was quantified in all groups and was significantly lower in both contaminated groups when compared to controls (Table 2). At the same time, only two of the three OH-fluorene metabolites analyzed were detected in the brain, namely the 9- and the 2-OH fluorene. The 9-OH fluorene was quantified in both groups and showed a significant increase only in the rats exposed to 150 ppb of fluorene (Table 2). In parallel, the 2-OH fluorene was not detected in controls, reached a level just above the limit of detection (0.10 ng/mL) in 1.5 ppb fluorene-exposed rats and was significantly higher in animals that inhaled 150 ppb of fluorene (Table 2).

### 4. Behavioral effects of fluorene exposure

#### 4.1. Elevated-plus maze

The total number of arms visited, the closed arm entries, and the open arm entries did not significantly differ among the groups (Table 3). When considering the time spent in the different parts of the maze, the fluorene-exposed rats showed a significant increase in the center time whereas the closed arm time remained the same. Thus, a 14–18% decrease in the time spent in the open arms was observed in the two fluorene-contaminated groups, but it was not significant (Table 3). In parallel, a non-significant 7–22% decrease in the number of head-dippings performed in the open area of the maze was observed whereas the total number of head-dipps remained the same between the groups. No significant differences were measured in the number of rears whatever the part of the maze considered.

**Table 3:** Effects of fluorene on anxiety-related behavior assessed in the elevated-plus maze.

Variable	Restrained Controls	1.5 ppb	150 ppb	ANOVA	
				F(2, 35)	*p*
Total arm entries	15.4±1.1	15.4±0.8	16.8±1.4	0.471	n.s.
Open arm entries	5.9±0.8	5.7±0.6	5.9±0.6	0.047	n.s.
Closed arm entries	9.5±0.7	9.8±0.9	10.8±0.9	0.713	n.s.
% Open arm entries	37.6±3.1	37.3±4.3	35.0±3.1	0.167	n.s.
Open arm time (s)	87.5±9.7	75.4±12.7	71.7±9.9	0.565	n.s.
Closed arm time (s)	148.7±11.2	147.8±14.8	148.8±10.7	0.998	n.s.
Central area time (s)	63.8±6.3	76.8±5.4 *	79.5±4.6 *	4.352	< 0.05
Total head-dipping	7.7±0.8	8.3±0.9	8.2±1.2	0.902	n.s.
% head-dipping in open arms	62.3±5.7	54.7±7.4	40.0±7.1	2.795	p = 0.08
Total rearing	22.5±1.5	22.3±2.0	25.3±1.2	0.357	n.s.
% rearing in closed arms	69.8±3.5	72.4±4.3	70.5±2.6	0.151	n.s.

Results are expressed as mean ± S.E.M. of n = 12 rats/group. Dunnett's t-test was used for multiple comparisons.

* *p*<0.05, statistical significant difference from controls. Abbreviations: n.s., not significant.

#### 4.2. Open-field

**Figure 1:**
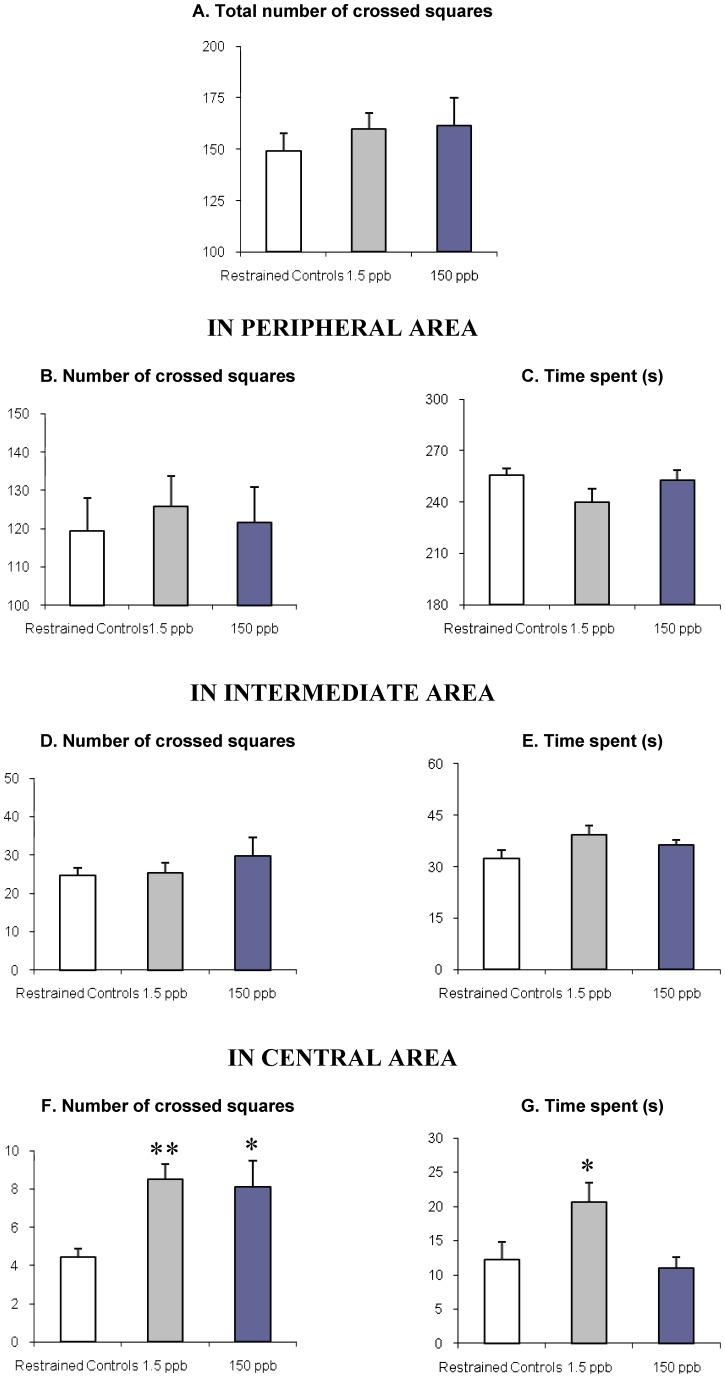
Effects of fluorene on locomotor activity and anxiety assessed in the open-field. Results are expressed as mean ± S.E.M. of n = 12 rats/group. * *p*<0.05, ** *p*<0.01, statistical significant differences from restrained control rats (Dunnett's *t*-test for multiple comparisons).

The locomotor activity assessed by the total number of crossed squares was not significantly modified by the fluorene exposure (Figure 1A) despite a slight increase of this variable by 7% and 9% in 1.5 and 150 ppb fluorene-exposed rats, respectively. This was related to the significant increase in the total number of crossed squares in the central area of the maze observed in both contaminated groups compared to the restrained control rats (Figure 1F). Moreover, animals exposed to 1.5 ppb of fluorene spent significantly more time in the same part of the maze compared to the control rats whereas the 150 ppb exposed-animals did not (Figure 1G).

#### 4.3. Y-maze

Animals exposed to 1.5 or 150 ppb of fluorene did not show any learning disabilities as reflected by the same percentage of spontaneous alternation in both groups (Table 4). Moreover, fluorene had no effect on the rat activity whatever the variable measured (the total number of arms visited, the number of arms visited per minute, or the number of arms visited during the first minute of testing) (Table 4).

**Table 4:** Effects of fluorene on spontaneous alternation performances assessed in the Y-maze.

Variable	Restrained Controls	1.5 ppb	150 ppb	ANOVA	
				F(2, 35)	*p*
% Spontaneous alternation	66.7±2.4	65.3±1.7	59.5±3.8	1.762	n.s.
Total arm entries	32.6±1.6	35.8±1.3	32.5±1.9	1.264	n.s.
Number of arms visited/min	3.3±0.2	3.6±0.1	3.3±0.2	1.264	n.s.
Number of arm entries (1st min)	5.1±0.3	5.5±0.3	5.4±0.2	0.389	n.s.

Results are expressed as mean ± S.E.M. of n = 12 rats/group. Abbreviations: n.s., not significant.

#### 4.4. Eight-arm maze

Control animals showed an ability to learn the topography of the maze, as indicated by a significant reduction in the total number of arm entries, and the concomitant increase in the number of arms visited before the first error over the 12 days of testing (Table 5). In parallel, the time needed for reaching all eight goal arms significantly decreased with a rise in the number of arms entered per min (Table 5). Similar behavioral performances were observed in fluorene-exposed animals whatever the concentration used (1.5 and 150 ppb), suggesting the inability of fluorene to impair the learning and memory capacities of the animals. This was confirmed by the lack of statistical differences among the 3 groups and the absence of statistical interactions between the group and the time of testing (Table 5).

**Table 5:** Effects of fluorene on spatial memory performances assessed in the eight-arm maze.

Group		Day 1	Day 4	Day 8	Day 12	Group of exposure		Time of testing			Group x Time	
						F(2, 35)	*p*	F(2, 35)	*p*		F(2, 35)	*p*
Total time (s)												
	Restrained Controls	129.7±10.6	86.2±9.6	68.7±4.5	76.1±8.0	1.345	n.s.	20.442	**		0.525	n.s.
	1.5 ppb	119.9±11.7	81.7±8.6	72.9±4.3	69.2±2.5							
	150 ppb	132.0±13.5	76.2±3.9	69.9±4.1	56.3±3.2							
Total arm entries												
	Restrained Controls	12.2±0.7	9.8±0.6	9.3±0.6	9.4±0.8	0.419	n.s.	12.396	**	0.323		n.s.
	1.5 ppb	12.3±1.3	9.8±0.6	9.1±0.3	8.5±0.2							
	150 ppb	12.6±1.1	10.2±0.6	9.3±0.5	8.3±0.2							
Arm entries before the 1st error												
	Restrained Controls	5.8±0.4	6.8±0.4	6.5±0.5	6.9±0.5	0.503	n.s.	11.51	**	0.91		n.s.
	1.5 ppb	5.4±0.6	6.7±0.4	7.2±0.2	7.3±0.3							
	150 ppb	4.5±0.6	6.2±0.5	7.4±0.3	6.8±0.6							
Number of arm visited/min												
	Restrained Controls	5.7±0.3	7.1±0.4	7.7±0.2	7.3±0.5	2.497	n.s.	22.901	**	0.388		n.s.
	1.5 ppb	5.9±0.6	7.3±0.4	8.0±0.3	7.6±0.1							
	150 ppb	6.1±0.5	8.1±0.4	8.1±0.3	9.3±0.2							

Results are expressed as mean ± S.E.M. of n = 12 rats/group. ** *p*<0.01, statistical significant variation (two-way ANOVA with repeated measures as one factor). Abbreviations: n.s., not significant.

### 5. Effects of the restraint stress

The body weight of the restrained control rats significantly decreased over the experimental period compared to the freely-moving animals (Table 6). Behavioral measurements of the activity also showed a significant effect of the exposure to this stressful situation as reflected by the significant increases in the total number of squares crossed in the open-field and the total number of arms visited in the Y-maze (Table 6). Despite that, corticosterone blood levels, brain, liver, and surrenal gland weights remained the same between the two groups (Table 6). In the same way, no effects of the restraint stress were observed on anxiety- and memory-related behaviors (data not shown).

**Table 6:** Effects of the restraint stress induced by the nose-only fluorene exposure device.

Variable	Freely-moving rats	Restrained controls
**Physiological endpoints**		
Body weight (g)		
* day before starting the habituation to the tubes*	243.22±2.01	246.89±2.48
* day before starting the exposure*	282.56±3.45	270.61±2.95 #
* after 7 days of exposure*	303.00±4.56	288.56±4.10 #
* after 14 days of exposure*	317.83±5.41	299.00±4.97 #
Relative brain weight (% b.w.)	0.59±0.01	0.60±0.03
Relative liver weight (% b.w.)	3.15±0.09	2.95±0.05
Relative surrenal gland weight (% b.w.)	0.013±0.002	0.020±0.003
Levels of corticosterone (ng/ml)		
* day before starting the habituation to the tubes*	299.14±32.42	248.55±22.20
* day before starting the exposure*	283.42±16.19	278.27±15.13
* after 7 days of exposure*	252.25±21.91	278.08±14.37
* after 14 days of exposure*	163.25±30.99	193.33±15.05
**Behavioral measurements**		
Total number of crossed cases in the open-field	117.8±10.5	149.2±8.1 #
Total arm entries in the Y-maze	25.3±1.1	32.6±1.6 ##

Results are expressed as mean ± S.E.M. of n = 12 rats/group. ## *p*<0.01, #*p*<0.05, statistical significant differences between freely-moving animals and restrained controls (Student t-test). Abbreviations: b.w., body weight.

## Discussion

The present study aimed to investigate the behavioral toxicity of fluorene, which is one of the most volatile PAHs, through repeated exposure of adult rats to a contaminated atmosphere in a nose-only model of inhalation. Whereas several studies investigated the levels of exposure in general and occupational environments, this work is probably the first one that investigates the toxicity of the inhalation of this compound through some behavioral investigation. The experimental design of the study is original by the model of exposure used (inhalation nose-only), the use of fluorene as a representative compound for the atmospheric pollution, the levels of exposure that were calculated as to correspond to different human situations of exposure, and the technical ability to generate the fluorene-contaminated atmosphere at the appropriate levels of contamination (1.5 and 150 ppb) and to ensure these levels of exposure through the monitoring of the contaminated atmosphere. This work is also the first one to report brain and blood concentrations of this compound after inhalation exposure, for which a specific GC-MS/MS analytical method has been developed [23]. Moreover, the study was designed to evaluate the possible influence of the stressful context of the model of exposure in the behavioral toxicity of fluorene.

### 1. Behavioral toxicity of fluorene

Regarding learning and memory performances, no behavioral modifications were observed in fluorene-exposed rats compared to controls, suggesting the inability of fluorene to impair the cognitive functions after 14 days of exposure. In comparison, BaP and 3-methylcholanthrene were shown to disrupt the learning and memory performances in adult animals [14], [29]. Therefore, the absence of learning disturbances observed for fluorene suggests a form of toxicity of this compound that is rather different from BaP or other PAHs. These differences may be related to multiple factors including both variations in molecular structure, ways of metabolism, toxicokinetic characteristics, brain targets of interactions, way of administration and levels of exposure. Whereas BaP has been demonstrated to modify several functional endpoints in the brain including neuronal excitability, oxidative stress, and several neurotransmitter systems [7], little is known about the brain cellular and/or molecular toxicity of fluorene, requiring the testing of the effects of this compound [30].

In parallel, the ability of fluorene to modulate the anxiety level of rats was tested in the open-field and in the elevated-plus maze, both of which are widely used in rodents to assess emotionality, anxiety and/or responses to stress [25], [26]. In the open-field, fluorene-exposed rats (1.5 and 150 ppb) were less anxious than controls, as reflected by the significant increase in the number of crossed squares in the central unprotected part of the maze (Figure 1F). Concomitantly, the time spent in the same area of the maze also increased in a significant way, but only in rats exposed to 1.5 ppb of fluorene (Figure 1G). The same reduction in anxiety was not observed in the elevated-plus maze in which the fluorene-treated rats showed a significant increase in the central place of the maze which is the zone of decision making [25], while the exploration of open arms slightly decreased (Table 3). Such discrepancies between the two mazes would probably be related to the more aversive feature of the elevated-plus maze compared to the open-field [31], and the ability of fluorene to slightly modulate the level of anxiety of the animals when exposed through the inhalation pathway. Results obtained in rats daily administered i.p. or p.o. with higher doses of fluorene (1, 10 or 100 mg/kg/day) for 28 days showed a significant reduction in the anxiety level measured in these two mazes (unpublished data). Finally, our results are consistent with the ones obtained with BaP in adult mice [14], and suggest the capacity of this family of pollutants to modulate the level of anxiety, depending on the molecule, the dose and the method of administration.

### 2. Effects of restraint stress

Whereas the results obtained in fluorene-exposed rats and presented above were compared with those of the appropriate restrained control animals, various behavioral and physiological markers indicative of the level of stress were measured in freely-moving rats inhaling non-contaminated air, and compared with those of the restrained animals in order to discriminate between the contribution of the stressful context of the model of exposure and the behavioral toxicity of fluorene. Thus, the results of the present study show a significant increase in the general activity of restrained animals (Table 6) whereas their level of anxiety and learning performances remained unaffected (data not shown). Contradictory results including no alterations, increase or decrease in the locomotor activity and the anxiety level of rats chronically exposed to restraint stress have been reported [32]–[34]. Such behavioral changes may be related to the adaptation of the hypothalamo-pituitary axis (HAP). Indeed, the HPA response can desensitize or remain stable especially when the same stressor is repeated [35]. Therefore, the corticosterone blood levels measured in the present study decreased over the period of exposure in both groups without any significant difference between the restrained animals and the freely-moving rats, suggesting a habituated corticosterone response despite the daily exposure to the restraint stress. These results are in accordance with those obtained by Narciso et al. (2003) [36] who concluded to the full adaptation of the animals to the restraint imposed by a nose-only inhalation device after 14 days of fixed-duration daily restraint. Taken together, all these results are indicative of a non-specific influence of the stressful context of the fluorene exposure on the general activity of all restrained animals (including both control and fluorene-exposed animals). However, significant behavioral disturbances were observed in fluorene-contaminated rats when compared with the appropriate restrained control animals, suggesting a specific behavioral toxicity of this compound.

### 3. Blood and brain concentrations of fluorene and mono-hydroxylated metabolites

Analyses of fluorene and monohydroxylated metabolites were performed in blood and brain samples in order to characterize the level of exposure of the animals to this contaminant. In blood, fluorene and its 9-OH metabolite were detected in the animals of the 4 experimental groups, suggesting the difficulty to overcome the influence of the environmental pollution. However, the environmental contamination of the control rats with fluorene does not seem to be related to our experimental design. Indeed, the monitoring of the atmosphere of the two control chambers showed the absence of fluorene in the air whereas the appropriate levels of concentration of fluorene were measured in parallel in the two chambers used to expose the animals (Table 1). In addition, continuous sampling of the air of the two control chambers for 3 days was performed (this corresponded to a filtered air volume of 4 m^3^) and did not allow the detection of fluorene. Considering a limit of detection of 0.8 µmol/L in acetonitrile, it is possible to assure that the level of concentration of the contaminant in the two control chambers was below 0.1 µg/m^3^ (0.015 ppb). Moreover, the analyses of PAHs were below the limit of detection of 1 ng/g for food and sawdust, and 10 ng/L for water, and confirmed the lack of contamination of the animals through these materials. Therefore, the fluorene blood levels reported in the controls may be considered as representative of the exposure of both groups of rats to an environmental background level of contamination. Indeed, fluorene is one of the most volatile PAHs and is consequently one of the most abundant in air [1], [5], [17]. In this way, the authors reported in a previous study, the contamination of rats during their stalling in supplier as the source of contamination [23].

Furthermore, the measurement of the two other metabolites, namely the 2- and the 3-OH fuorenes, only in blood of both fluorene-exposed rats clearly demonstrated the exposure of such animals to this compound. 2-OH fluorene, which is established as a biomarker of exposure to fluorene [37], [38], was detected in both exposed groups in correlation with the fluorene level monitored in the air of the two contaminated chambers. Thus, a detectable concentration of this metabolite was measured in blood of 1.5 ppb-exposed rats that was 100 times higher in the animals exposed to the highest dose (150 ppb, Table 2). The same thing was observed for 3-OH fluorene with a 50-fold increase in its blood concentration between the two levels of fluorene exposure. In addition, an unidentified metabolite was also detected in both groups of rats exposed to this contaminant with a similar increase in its concentration. Taken together, these results clearly demonstrated the dose-related exposure of the animals to increasing controlled levels of fluorene in air.

No significant differences were observed between the four groups regarding the blood fluorene level despite the decrease observed in 1.5 ppb- and 150 ppb-exposed rats (−20% and −50%, respectively). The discrepancies between the results expected and those obtained may be accounted for by metabolism efficiency. The concomitant increase in the concentrations of various metabolites with the atmospheric level of fluorene suggests that most of the compound inhaled could be rapidly metabolized in peripheral organs such as liver or lung. This assumption seems to be confirmed by the cerebral levels of fluorene that were significantly lower in the two contaminated groups compared to controls (Table 2). In control rats, the unmetabolized fluorene may firstly diffuse through the brain capillary endothelial cells, penetrate the brain and then be slowly accumulated in situ [39]. In fluorene-exposed animals, the blood concentration of fluorene may reach a sufficient level to induce the metabolism with a reduction in its brain concentration and the lack of increase in its blood concentration as consequences. This phenomenon is coupled with the detection and the increase in concentrations of monohydroxylated fluorene in brain. For example, the brain 2-OH fluorene level was non detected in controls, just above the limit of detection in 1.5 ppb-exposed animals, and significantly exceeded it when submitted to the highest level of exposure (150 ppb). In parallel, the brain concentration level of the 9-OH fluorene remained the same between both control groups of rats and the animals contaminated with 1.5 ppb of fluorene, and significantly increased in animals exposed to 150 ppb of fluorene. Moreover, these results are in accordance with studies which have dealt with PAH peripheral metabolism [40], all of them suggesting a role for peripheral metabolic pathways in the generation of several mono- and di-hydroxylated metabolites which could accumulate in the cerebral tissue in addition to the metabolites produced within the brain [14].

## Conclusions

The present results show the ability of a 14-day inhalation of fluorene at environmental and occupational levels of exposure to induce some slight behavioral disturbances related to anxiety whereas the learning and memory capacities remained unaffected. Significant levels of fluorene and several monohydroxylated metabolites were detected in the blood and the brain of the same animals, showing the ability of this compound to be metabolized and to reach the cerebral compartment. Thus, the behavioral disturbances observed between the control rats and the fluorene-exposed animals suggest a role for both the parent compound and its metabolites in the brain toxicity of this pollutant. At final, the present study contributes to demonstrate the susceptibility of the adult brain exposed to volatile PAHs in general and fluorene in particular.

## References

[1] Ramirez N, Cuadras A, Rovira E, Marce RM, Borrull F (2011) Risk assessment related to atmospheric polycyclic aromatic hydrocarbons in gas and particle phases near industrial sites. Environ Health Perspect 119: 1110–1116. 21478082 10.1289/ehp.1002855 PMC3237345

[2] Srogy K (2007) monitoring of environmental exposure to polycyclic hydrocarbons: A review. Environ Chem Lett 5: 169–195. 29033701 10.1007/s10311-007-0095-0 PMC5614912

[3] Boffetta P, Jourenkova N, Gustavsson P (1997) Cancer risk from occpational and environmental exposure to polycyclic aromatic hydrocarbons. Cancer Causes Control 8: 444–472. 9498904 10.1023/a:1018465507029

[4] Unwin J, Cocker J, Scobbie E, Chambers H (2006) An assessment of occupational exposure to polycyclic aromatic hydrocarbons in the UK. Ann Occup Hyg 50: 395–403. 16551675 10.1093/annhyg/mel010

[5] IARC (2010) Working Group on the Evaluation of Carcinogenic Risks to Humans. Some Non-heterocyclic Polycyclic Aromatic Hydrocarbon Related Industrial Exposure. IARC monographs on the evaluation of carcinogenic risks to humans. Lyon: International Agency for research on Cancer 92.

[6] Santodonato J (1997) Review of the estrogenic and antiestrogenic activity of polycyclic aromatic hydrocarbons: relationship to carcinogenicity. Chemosphere 34: 835–848. 9569946 10.1016/s0045-6535(97)00012-x

[7] Schroeder H (2011) Developmental brain and behaviour toxicity of air pollutants. A focus on the effects of Polycyclic Aromatic hydrocarbons (PAHs). Crit Rev Environ Sci Technol 41: 2026–2047.

[8] Dayal H, Gupta S, Trieff N, Maierson D, Reich D (1995) Symptom clusters in a community with chronic exposure to chemicals in two superfund sites. Arch Environ Health 50: 108–111. 7786046 10.1080/00039896.1995.9940887

[9] Kilburn KH, Warshaw RH (1995) Neurotoxic effects from residential exposure to chemicals from an oil reprocessing facility and superfund site. Neurotoxicol Teratol 17: 89–102. 7760780 10.1016/0892-0362(94)00057-k

[10] Majchrzak R, Sroczynski J, Chelmecka E (1990) Evaluation of the nervous system in workers in the furnace and coal divisions of the coke producing plants. Med Pr 41: 108–113. 2215199

[11] Niu Q, Zhang H, Li X, Li M (2010) Benzo[a]pyrene-induced neurobehavioral function and neurotransmitter alterations in coke oven workers. Occup Environ Med 67: 444–448. 19854696 10.1136/oem.2009.047969

[12] Saunders CR, Ramesh A, Shockley DC (2002) Modulation of neurotoxic behavior in F-344 rats by temporal disposition of benzo(a)pyrene. Toxicol Lett 129: 33–45. 11879972 10.1016/s0378-4274(01)00467-2

[13] Saunders CR, Shockley DC, Knuckles ME (2003) Fluoranthene-induced neurobehavioral toxicity in F–344 rats. Int J Toxicol 22: 263–276. 12933321 10.1080/10915810305114

[14] Grova N, Schroeder H, Farinelle S, Prodhomme E, Valley A, et al (2008) Sub-acute administration of benzo[a]pyrene (B[a]P) reduces anxiety-related behaviour in adult mice and modulates regional expression of N-methyl-D-aspartate (NMDA) receptors genes in relevant brain regions. Chemosphere 73: S295–302. 18442843 10.1016/j.chemosphere.2007.12.037

[15] Grova N, Valley A, Turner JD, Morel A, Muller CP, et al (2007) Modulation of behavior and NMDA-R1 gene mRNA expression in adult female mice after sub-acute administration of benzo(a)pyrene. Neurotoxicology 28: 630–636. 17397927 10.1016/j.neuro.2007.01.010

[16] Wormley DD, Chirwa S, Nayyar T, Wu J, Johnson S, et al (2004) Inhaled benzo(a)pyrene impairs long-term potentiation in the F1 generation rat dentate gyrus. Cell Mol Biol (Noisy-le-grand) 50: 715–721. 15641162

[17] Li Z, Mulholland JA, Romanoff LC, Pittman EN, Trinidad DA, et al (2010) Assessment of non-occupational exposure to polycyclic aromatic hydrocarbons through personal air sampling and urinary biomonitoring. J Environ Monit 12: 1110–1118. 21491629 10.1039/c000689k

[18] Gmeiner G, Stehlik G, Taush H (1997) Determination of seventeen polycyclic aromatic hydrocarbons in tobacco smoke condensate. J Chromatogr A 767: 163–169.

[19] Kot-Wasik A (2004) Studies on fluorene stability in different liquid media. Anal Chem Acta 505: 289–299.

[20] U.S. Environmental Protection Agency (2002) Latest findings on national Air quality: 2000. Status and trends.

[21] OECD (2009) Guideline for the testing of chemicals. Section 4: Health effect/test No. 403: Acute Inhalation Toxicity.

[22] Directive EU (2010) 2010/63/EU of september 2010 on the Approximation of Laws. regulations and administrative provisions of the member states regarding the protection of animals used for experimental and other scientific purposes. 20397315

[23] Grova N, Salquebre G, Schroeder H, Appenzeller BM (2011) Determination of PAHs and OH-PAHs in rat brain by gas chromatography tandem (triple quadrupole) mass spectrometry. Chem Res Toxicol 24: 1653–1667. 21916506 10.1021/tx2003596

[24] Ling S, Jamali F (2003) Effect of cannulation surgery and restraint stress on the plasma corticosterone concentration in the rat: application of an improved corticosterone HPLC assay. J Pharm Pharm Sci 6: 246–251. 12935437

[25] Violle N, Balandras F, Le Roux Y, Desor D, Schroeder H (2009) Variations in illumination, closed wall transparency and/or extramaze space influence both baseline anxiety and response to diazepam in the rat elevated plus-maze. Behav Brain Res 203: 35–42. 19389429 10.1016/j.bbr.2009.04.015

[26] Gould TD, Dao TD, Kovacsics CE (2009) The open field test. In: Mood and Anxiety Related Phenotypes in Mice: Characterization Using Behavioral Tests. Gould TD, editor. Neuromethods vol. 42, Springer Protocols, 1–20.

[27] Hugue RN (2004) The value of spontaneous alternation behavior (SAB) as a test of retention in pharmacological investigation of memory. Neurosci Biobehav Rev 28: 497–505. 15465137 10.1016/j.neubiorev.2004.06.006

[28] Blaise SA, Nedelec E, Alberto JM, Schroeder H, Audonnet S, et al (2009) Short hypoxia could attenuate the adverse effects of hyperhomocysteinemia on the developing rat brain by inducing neurogenesis. Exp Neurol 216: 231–238. 19124018 10.1016/j.expneurol.2008.11.020

[29] Konstandi M, Pappas P, Johnson E, Lecklin A, Marselos M (1997) Suppression of the acquisition of conditioned avoidance behavior in the rat by 3-methylcholanthrene. Pharmacol Biochem Behav 56: 637–641. 9130288 10.1016/s0091-3057(96)00407-8

[30] Grandjean P, Landrigan PJ (2006) Developmental neurotoxicity of industrial chemicals. Lancet 368: 2167–2178. 17174709 10.1016/S0140-6736(06)69665-7

[31] Carola V, D'Olimpio F, Brunamonti E, Mangia F, Renzi P (2002) Evaluation of the elevated plus-maze and open-field tests for the assessment of anxiety-related behaviour in inbred mice. Behav Brain Res 134: 49–57. 12191791 10.1016/s0166-4328(01)00452-1

[32] Bowman RE, Ferguson D, Luine VN (2002) Effects of chronic restraint stress and estradiol on open field activity, spatial memory, and monoaminergic neurotransmitters in ovariectomized rats. Neuroscience 113: 401–410. 12127097 10.1016/s0306-4522(02)00156-2

[33] Dubovicky M, Jezova D (2004) Effect of chronic emotional stress on habituation processes in open field in adult rats. Ann N Y Acad Sci 1018: 199–206. 15240369 10.1196/annals.1296.023

[34] Gregus A, Wintink AJ, Davis AC, Kalynchuk LE (2005) Effect of repeated corticosterone injections and restraint stress on anxiety and depression-like behavior in male rats. Behav Brain Res 156: 105–114. 15474655 10.1016/j.bbr.2004.05.013

[35] Aguilera G (1998) Corticotropin realisng hormone, receptor regulation and the stress response. Trends Endocrinol Metabol: 329–336. 10.1016/s1043-2760(98)00079-4 18406298

[36] Narciso SP, Nadziejko E, Chen LC, Gordon T, Nadziejko C (2003) Adaptation to stress induced by restraining rats and mice in nose-only inhalation holders. Inhal Toxicol 15: 1133–1143. 12955618 10.1080/08958370390228592

[37] Amorim LC, Dimandja JM, Cardeal Zde L (2009) Analysis of hydroxylated polycyclic aromatic hydrocarbons in urine using comprehensive two-dimensional gas chromatography with a flame ionization detector. J Chromatogr A 1216: 2900–2904. 19038392 10.1016/j.chroma.2008.11.012

[38] Xia Y, Han Y, Zhu P, Wang S, Gu A, et al (2009) Relation between urinary metabolites of polycyclic aromatic hydrocarbons and human semen quality. Environ Sci Technol 43: 4567–4573. 19603678 10.1021/es9000642

[39] Ravindranath V, Bhamre S, Bhagwat SV, Anandatheerthavarada HK, Shankar SK, et al (1995) Xenobiotic metabolism in brain. Toxicol Lett 82–83: 633–638. 10.1016/0378-4274(95)03508-7 8597120

[40] Shimada T (2006) Xenobiotic-metabolizing enzymes involved in activation and detoxification of carcinogenic polycyclic aromatic hydrocarbons. Drug Metab Pharmacokinet 21: 257–276. 16946553 10.2133/dmpk.21.257

